# Fault Handling in Industry 4.0: Definition, Process and Applications

**DOI:** 10.3390/s22062205

**Published:** 2022-03-12

**Authors:** Heiko Webert, Tamara Döß, Lukas Kaupp, Stephan Simons

**Affiliations:** 1 Department of Electrical Engineering and Information Technology, Darmstadt University of Applied Sciences, Haardtring 100, 64295 Darmstadt, Germany; stephan.simons@h-da.de; 2 Department of Mathematics and Natural Sciences, Darmstadt University of Applied Sciences, Haardtring 100, 64295 Darmstadt, Germany; tamara.doess@stud.h-da.de; 3 Department of Computer Science, Darmstadt University of Applied Sciences, Haardtring 100, 64295 Darmstadt, Germany; lukas.kaupp@h-da.de

**Keywords:** cyber-physical systems, cyber-physical production systems, fault detection, fault classification, fault prioritization, fault amendment, fault modes, machine learning, FMEA

## Abstract

The increase of productivity and decrease of production loss is an important goal for modern industry to stay economically competitive. For that, efficient fault management and quick amendment of faults in production lines are needed. The prioritization of faults accelerates the fault amendment process but depends on preceding fault detection and classification. Data-driven methods can support fault management. The increasing usage of sensors to monitor machine health status in production lines leads to large amounts of data and high complexity. Machine Learning methods exploit this data to support fault management. This paper reviews literature that presents methods for several steps of fault management and provides an overview of requirements for fault handling and methods for fault detection, fault classification, and fault prioritization, as well as their prerequisites. The paper shows that fault prioritization lacks research about available learning methods and underlines that expert opinions are needed.

## 1. Introduction

For the manufacturing industry, a primary aim is to increase the productivity and quality alongside the reduction of unplanned downtimes of machines in production lines to be able for economic competition [1,2]. Machine downtime can be reduced by implementing predictive maintenance methods that will lead to operators taking care of machines that will soon fall into a fault mode. Still, machine faults occur, which could result in the necessity of replacing parts of the equipment. This may also lead to accidents and system failures that will cost millions in lost production, or pollution [3]. Therefore, proper fault handling is needed, as faults significantly impact reducing downtime and manufacturing costs. This process depends heavily on how early a fault is detected and identified after the occurrence, as more possibilities to act will exist [4]. In addition, there is also an impact on the meantime to repair (MTTR), which describes the average time until the machine has been repaired by operating personnel [5]. Fault detection includes the correct determination of the faults’ nature, their impact, and location in the manufacturing process, based on data produced by the manufacturing system [6,7]. The identified faults can be classified and then prioritized to accelerate the repair actions by the personnel. Therefore, supporting methods for the fault handling steps are needed [8]. Those depend on collected data that gives information about the machine’s status.

This data can be produced by sensors that are ubiquitous in modern manufacturing sites. Here, we focus on cyber-physical systems (CPS) in Industry 4.0, which is also known as cyber-physical production systems (CPPS) [9]. A CPS consists of both cyber-elements, e.g., software-modules and physical-components, e.g., sensors and actuators [6,10] and their interaction [11,12]. Regarding the distribution of the term CPPS in research, we also use the broader term of a CPS focusing on production systems. Complexity in fault detection rises in such an environment and, in addition to that, the probability of fault. In such complex systems, the probability of faults is higher [6,13], which leads to a more complicated and computationally intensive fault detection [12]. Therefore, the need for automated and scaleable fault handling methods gets even higher. In this paper, a fault will be defined as a machine state in which a process does not perform as required or needed (e.g., [12,14,15]).

This paper will review methods used for fault handling in manufacturing processes and will focus on their use in cyber-physical systems. To do so the concept fault handling in CPSs is introduced and requirements needed for fault handling methods are listed in Section 2. Several methods for the single steps of fault handling, namely fault detection (Section 3.1), fault classification (Section 3.2) and fault prioritization (Section 3.3), will be introduced shortly and a listing of literature references for each step will be given in Section 3. The results of the literature review will be discussed in Section 4. A conclusion and a prospect of future research areas will be given in Section 5.

## 2. Requirements for Effective Fault Handling

To perform effective fault handling in an industrial production environment, prerequisites are required to enable methods to perform well. First, there are the conditions that need to be fulfilled by the production site. Most fault diagnosis methods depend on the machines’ historical data within production lines. These methods are data-driven and can only perform well if a necessary amount of data is available [16]. Therefore, they need to be scalable to handle the huge amount of data. As modern production sites implement Internet of Things (IoT) devices [17] and several sensors to monitor machine condition, the data amount can also reach a high complexity. Sensors monitor various variables, based on both physics, like vibration, temperature, pressure, and based on processes of a manufacturing system, like process deviations, control settings, and machine specifications [1,17]. The complexity in data can be handled, e.g., via dimensionality reduction methods. Complexity is not only caused by the number of used sensors but also by the general structure of the machines in production lines and how these with their respective sensors are connected [5]. Additionally, the collected data is temporal. Sensor data is often gathered as time-series data, which also needs to be handled by methods that are used to produce insights about the machine’s status [18]. The temporal aspect is also relevant in industries that need to implement real-time fault detection and diagnosis as they can only use methods that are able to process data quickly and give results in real-time. Another general requirement for fault handling methods is the ability to train models on imbalanced data as in production, the amount of data that represents normal condition is more often seen as data that indicates faulty machine states [19,20].

In addition, data quantity and intrinsic complexity are a requirement for fault handling methods. CPSs produce a huge amount of data as they include many sensors and devices for processing and communication tasks, which connect physical elements like machines with cyber-physical data-processing units [6,11]. In addition, they consist of several subsystems, which leads to a higher complexity of the production line itself and its data relations [12,14]. Moreover, the higher complexity makes CPSs more prone to faults [13]. Therefore, fault handling supported by automated methods gets more important. The complexity and high dimensionality also result in computationally expensive feature extraction, which emphasizes the need for effective dimensionality reduction. Another reason for the need of automated fault handling in CPS is that not only the machines can experience fault states, but also the sensors themselves can generate faulty data, so sensor faults need to be handled as well.

As this paper reviews methods for different steps of fault handling, requirements for those steps need to be explicitly outlined. First, fault detection, which is the entry point of fault handling, works with the raw data of sensors. Therefore, the used methods need to be able to work with the given raw data, or feature extraction methods need to be implemented as well. Which method will be applied also depends on the raw data format that is available in the monitored system. Methods used for fault classification need to discriminate several groups of faults. A prerequisite to achieving this goal is that fault types are already defined, and historical data is labeled to train performant classifiers. In case labeling data is not feasible, then unsupervised methods are used. Therefore, experts need to be at hand to describe the fault groups former identified by the algorithm [1]. The classification and detection of faults with their respective types in the data are essential for the next step—fault prioritization. During the step of prioritization, the opinions of experts are needed [21,22]. They need to consider various faults that can occur in the monitored system. They also need to consider that machines do not work separately but depend on each other. So, in addition to the single machines’ criticality, experts need to take the chain of faults into account and how machines influence other processes in the production line [5]. Domain knowledge of experts is also important. Production lines and the employed CPSs have a high level of individuality with many configuration possibilities [23]. So copying methods from one production line to another is no feasible option without substantial manual effort.

## 3. Methods

**Figure 1:**
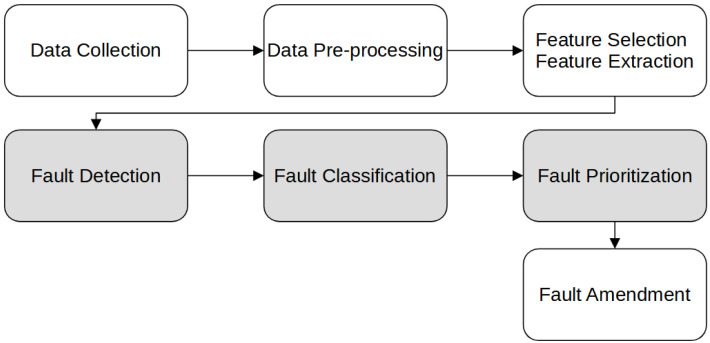
The overall fault handling process begins with the data collection, including pre-processing of the data and feature handling before training models for fault detection and fault classification. Afterwards, fault prioritization occurs; after that, all found faults will be handled manually by the operators’ personnel or automatically during the fault amendment process. Due to the individuality nature of the steps or their maturity, not all research fields are covered by this survey. Investigated research fields are covered by grey boxes, whereas white boxes cover the latter.

This section will show which methods are used in the literature for the different fault handling steps. The fault handling process, as shown in Figure 1, consists of the data collection, data pre-processing, and feature selection before training any models. The step of fault amendment is also designated after the decision has been made of which fault to tackle first. The methods presented in this paper are used for fault detection, fault classification, and fault prioritization steps. We focus on the mid-tier process (fault detection, fault classification, fault prioritization) because the data structure of each employed CPS is unique, and a generalization cannot be given for all available methods in the context of this work.

In addition, we declare fault amendment as an important process step, which describes the correction process after the fault handling steps. First attempts for fault amendment exists, e.g., Diedrich, Balzereit et al. [24,25,26] investigate an automated reconfiguration of CPSs after a fault is detected. To the best of our knowledge, no other automated attempts are made in the field of fault amendment but prompt us to include this step separately in the process without deeper analysis, leaving a field open for future research.

Therefore, this review only considers papers whose described methods have been mapped to one sub-step of the above-mentioned fault handling process (fault detection, classification, and prioritization). Note that various types and architectures exist for each listed method, which may be used in the context of Industry 4.0-related fault diagnosis. For this reason, we only provide a selected excerpt, which should be seen as examples for the following methods.

### 3.1. Fault Detection

Fault detection is the process of finding an occurrence of a fault in a unit of the monitored process based on measurements that are provided by the system. Those faults lead to abnormal or system-critical behavior of the machine, reducing the performance of the whole system significantly [21,27,28]. Some referenced papers also include the identification of further fault characteristics like impact, location, or time of occurrence and the actions taken to avoid further damage in the process of fault detection [6,7,29], whereas others state that this is a specific part of fault isolation [28]. In this paper, we consider methods that detect whether a fault occurred or not, despite a possible subdivision into the fault categories [20]: abrupt (e.g., [30]), incipient (e.g., [31]), or intermittent (e.g., [32]). Methods of fault classification will handle any further identification.

Fault detection methods can be separated into three groups [14,20,33]: Data-driven models learn the systems behaviour by training and thus depend on enough available data [16]. Data-driven approaches use analytical models and historical data. The approaches do not depend on knowledge of the monitored process structures and are scalable regarding the number of sensors they draw data from [22]. As the amount of gathered sensor data is increasing and machine learning techniques have been developed rapidly, many researchers focus on data-driven methods for fault diagnosis problems [34]. Model-based methods require building a specific model that includes the architecture and process of the monitored systems, as well as correlation and relations between the various process variables [20]. Mining those relations can be computationally intensive in large-scale systems with complex structures, and requires a huge amount of sensors [22]. Knowledge-based methods for fault diagnosis rely on sets of rules that are formed by expert knowledge of the monitored system and the relations between several fault types [33]. The dependency on knowledge makes those models very system-specific, which makes updating more complicated [16].

We align our work with recent outstanding surveys [14,20,35] and set the perspective around our defined fault handling process. Due to the scalability to vast amounts of data, this paper will focus on data-driven methods. Table 1 gives an overview of the presented methods, including further references, which have not been presented in this paper due to brevity.

#### 3.1.1. Neural Networks

Neural networks can be used as supervised or unsupervised learning techniques for the problem of fault detection [36,37]. Artificial neural networks (ANNs) learn complex non-linear functions. They also learn the importance of input features so that no preceding feature extraction methods are needed to reduce the complexity. However, input features need to be normalized to ensure that features with a larger scale will not be privileged [37].

Heo and Lee [37] applied a supervised ANN method to solve fault detection formulated as a binary classification problem. The model consists of an input layer with one node for each feature, several hidden layers, and one softmax layer that calculates the output values. The output neuron with the highest assigned value defines the data set class, which is in case of fault detection either normal or faulty. The authors train ANNs with different hidden layers and nodes per hidden layer using the rectified linear unit (ReLU) as an activation function. They apply the classifier to the Tennessee Eastman process, which is used as a benchmark process with defined fault types in literature (e.g., [49,50,51]). Their results are compared to those of Yin et al. [52] and Zhang and Zhao [53] and achieve the best overall detection rate.

Von Birgelen et al. [36] train self-organizing maps (SOM), as introduced by Kohonen [54], for fault diagnosis. SOM learns the characteristics of the normal behavior of components in a CPS. That means no faulty data sets are needed for training, which makes this method suitable for imbalanced data. SOM is an unsupervised neural network architecture that organizes its neurons in a topological map. At the end of the training stage, each neuron represents a unit, encompassing a part of the training data. Live data is mapped to the best fitting unit to calculate the quantization error, the distance between actual data value, and the mapped unit. If the quantization error exceeds a threshold, the data set is considered faulty, and further fault diagnosis can be performed. The authors evaluate their approach via experiments on real-world systems, including industrial plants.

#### 3.1.2. Random Forests

Random forests consist of uncorrelated decision trees trained independently and with a random choice of considered split features and training data sets. A random forest is a supervised method, as labeled data is needed to find splits [55]. Imbalanced data can be handled by weighted sampling methods or penalty on the misclassification of the minority class [56,57]. Due to the randomly selected split features and the independently trained trees, random forests are suitable for high dimensional data, and big data sets [58].

Yan and Zhou [39] use historical flight sensor data to detect and predict anomalies in aircraft components. They formulate the detection problem as a three-class classification problem with one class to represent normal state and two classes representing faults. A random forest is trained based on features that are extracted by using statistical analysis and correlation analysis. Their proposed method is evaluated in a case study on a component of an aircraft system.

#### 3.1.3. k-Nearest Neighbors (kNN) and Naïve Bayes

Both k-Nearest Neighbors and Naïve Bayes can be used as ensemble classifiers for fault detection in which the classifier chooses the class that is represented most among the results of trained classifiers. kNN is a non-parametric classification algorithm that classifies a new observation to the majority class among its k-Nearest Neighbors observations. The method is sensitive to non-informative features, which likely occur in high-dimensional data. The ensemble method with a random selection of features counters this problem [59]. The Naïve Bayes classifier is a probabilistic classifier that assumes that all features are pairwise independent. A new observation will be assigned to the class with the highest calculated posterior probability [60]. In comparison to a classical Bayes approach, the ensemble method results in higher efficiency of Naïve Bayes with regard to high-dimensional data, because in the classification stage, all features are considered to calculate the posterior probability [61].

Fan et al. [40] use ensemble models based on k-Nearest Neighbors (kNN) and Naïve Bayes classifiers to classify wafers of semiconductor manufacturing as flawless or faulty. They train a random forest model to handle high-dimensional data to get the variable importance of all sensor variables. K-means cluster those, and the cluster variables with the highest average variable importance are used in the final fault detection step. Ensemble models based on kNN and Naïve Bayes were trained with data that included an equal amount of randomly selected faulty and normal data sets to prevent inaccuracy due to class imbalance. The models were compared by sensitivity and specificity, and the kNN ensemble method performed better than the Naïve Bayes method.

#### 3.1.4. Kernel Principal Component Analysis

Kernel Principal Component Analysis (KPCA) is a self-supervised learning method that can be used to detect faulty observations. KPCA is an expansion of PCA where the data points are mapped into a higher-dimensional space by a kernel function. Then, PCA is performed in the higher-dimensional space [62]. During the training process, KPCA is performed on data samples that represent the normal state; hence, class imbalance in the case of fault detection is irrelevant. Consequently, KPCA succeeds on imbalanced and only partly labeled production line data. The maximum reconstruction error during the training stage defines the threshold used for fault detection in the test stage. For new data sets, the reconstruction error is calculated, and in case the threshold exceeds, the data set is considered faulty. As the kernel is an N by N matrix, with N being the number of observations, the time to generate the kernel matrix strongly increases if N is high. The KPCA method for fault detection is used by Wang et al. [19]. They evaluate their method in a case study based on experiments on a data set comprising industrial etching processes for fault detection. Yang, Chen, and Sun [41] meet the problem that KPCA cannot be used for real-time detection. This has been possible by reducing the training data set with an approximate basis that consists of a minimum of training samples but still represents the total training samples well.

#### 3.1.5. Typicality and Eccentricity Data Analysis

Typicality and Eccentricity Data Analysis (TEDA) is an unsupervised method introduced by Angelov [44] that can be used for fault detection, as well as classification, clustering, and prediction problems [43,63]. The model uses a data analysis method, following the concepts of typicality and eccentricity. In this context, typicality is described as the spatial similarity of a data sample to all other data samples, whereas eccentricity states the difference of a data sample from all other data samples. The method operates without any assumptions about the data distribution and data independence, which is unlikely in real-world scenarios. In addition, TEDA is a recursive algorithm, which makes this method fast and suitable for big data and real-time applications, resulting in a low computational complexity. Typicality and eccentricity are recalculated with every new data sample, so a threshold needs to be defined that separates normal from outlier (faulty) data samples [35].

Bezerra et al. [3] and Costa et al. [43] use TEDA as a fully autonomous algorithm for fault detection in industrial processes. Several signals are used to detect data samples as faulty. With the TEDA method, no prior knowledge of the processes and data samples and no user-defined parameters are needed. The TEDA method was used as an unsupervised learning algorithm in Lou and Li [45] by selecting features via the Laplacian Score method before training to make a priori knowledge during pre-processing stage negligible.

#### 3.1.6. Improved Support Vector Machines

Improved Support Vector Machines (SVM) for fault detection, named online sparse least squares SVM (OS-LSSVM), is proposed by Deng et al. [46]. They use the method for the detection and prediction of sensor faults. The sensors produce time-series data, which is analyzed based on sliding windows. The approach is based on the LSSVM method introduced by Suykens et al. [64]. Additionally, a sparsity component is implemented, which states that all input vectors can be linearly represented by the base vector space so that all training samples can be replaced by the base vector set. Furthermore, the training data is acquired with the sliding time window method that only considers the latest data points. This increases the prediction speed so that the method can be used for the real-time prediction of faults. For evaluation, the proposed method is applied on a gyro sensor. The results show that the residual error is lower when using LSSVM without the proposed sparsity component, while the forecasting time decreases with the sparsity component.

### 3.2. Fault Classification

In this paper, the fault classification process includes the fault detection of various fault types by clustering analysis and the classification of detected faults into predefined fault classes. In both cases, data that indicates faults need to be analyzed by experts with deep knowledge of the monitored system. In the case of clustering, experts need to define which identified cluster represents which fault type (unsupervised learning). In the case of classification, experts need to define which fault types can occur in the monitored system and which historical data sets represent those fault types (supervised learning).

Fault classification and therefore the generalization of faults is a prerequisite for fault prioritization (Section 3.3) thus experts can focus on a limited number of identified fault types. An overview of the presented fault classification methods is given in Table 2.

#### 3.2.1. Fault Clustering Methods

Fault clustering methods include k-means clustering, Gaussian-Mixture-Model clustering, and fuzzy-c-means clustering. Those methods are used by Amruthnath and Gupta [1,2] to identify clusters in vibration data of a rotating fan in different setups. By using unsupervised learning techniques, they address the challenges of supervised learning for early fault detection, such as the necessity of historical, labeled data and the incapability of classifying new faults that are not known at training time, which results in an extended training time and an inflexible model. On the contrary, unsupervised learning methods can be used for class structures, in which no knowledge of the original data is required. Both papers use PCA for dimensionality reduction and make the assumption that vibration is the only significant feature.

Gaussian-Mixture-Model is a non-parametric density estimation method that can identify several Gaussian distributions within a data set. Each of the different distributions represents a cluster. Amruthnath and Gupta [1] identified six clusters in their data with the Gaussian Mixture Model method in total, of which three represent redundant healthy states of the machine, as well as one for each of the following faulty states: operating failure, equipment failure and total shutdown of the machine. Experts defined the representations of the clusters. For the k-means method, the silhouette method identified an optimal number of two clusters. The clusters represent healthy and faulty states in general, but differentiation between operating failure, equipment failure, and total shut-off is impossible. In another research study, Amruthnath and Gupta [2] identified five clusters with the Gaussian-Mixture-Model method. Based on the elbow method, three clusters were identified with k-means, representing the healthy state, warning, and faulty state. Additionally, the fuzzy-c-means method was used. Fuzzy-c-means clustering is an extension of k-means clustering, improved by Bezdek [81]. The parameter *c* is comparable to the one *k* of k-means. The fuzzy aspect is given because all data sets belong to every cluster with a certain weight depending on its distance to the cluster’s centroid. The fuzzy-c-means method achieves the same results as the k-means method if hierarchical clustering has been performed.

#### 3.2.2. Neural Networks

Neural networks and their variants can be used for fault classification. Heo et al. [37] propose an artificial neural network (ANN) model for classifying data into 17 fault types for the Tennessee Eastman process. Normal and faulty data are used as training and test data. An ANN with three hidden layers using both ReLU activation function and a softmax layer with softmax function is compared to results of two references [49,82] and achieves the best overall classification rate for the selected fault types and normal state. The authors explain the better results with their network design so that the ANN performs fault detection and classification simultaneously.

Another ANN is the autoencoder. An autoencoder is one kind of unsupervised learning technique that identifies important features in the input data. The model encodes the input data into a lower-dimensional space and tries reconstruction through decoding. The difference between original input data and reconstructed output data gives the reconstruction error, which will be minimized by training the autoencoder [83]. Several methods encode the input into a lower-dimensional space.

Lv et al. [49] apply a stacked sparse autoencoder with a softmax classifier to the multi-class classification problem of several fault types and normal mode. A stacked sparse autoencoder is a neural network consisting of several sparse autoencoders. The sparsity penalty causes most of the hidden layer units not to get activated and thus focus on unique features that identify the classes of the training data [83]. The model is evaluated on data of the Tennessee Eastman Process experiment that got detected as faulty beforehand and achieved the best average fault classification rate compared to other state-of-the-art approaches, including sparse representation, random forest, SVM, and structural SVM.

Fang et al. [66] propose an autoencoder to detect 10 different fault types from data generated in a satellite power system. The neural network includes two hidden layers that use the de-noising autoencoder method. The input layer consists of 48 nodes for each input parameter. The output layer consists of 10 output nodes for each fault type to be identified. The proposed method is compared to the deep belief network and deep Boltzmann machine methods based on the same data set. The results indicate that the proposed deep neural network method performs best.

Another neural network variant is the convolutional neural network (CNN). Goodfellow, Bengio, and Courville [83] describe CNNs as neural networks used for processing data with grid-like topology, like time-series data. A CNN includes at least one convolutional layer as a hidden layer. A convolutional layer transforms input data into sparse representations by sliding a minimum of one kernel matrix over the input data matrix and calculating the dot product in each step. This sparse representation will be used as input to the next network layer. The last layer of the CNN is a fully connected classification layer that uses the softmax function. CNN’s merge the feature extraction step and model training step, which saves computational time so that CNNs can be used in real-time fault classification applications. This has been declared by Ince et al. [71], who use a one-dimensional CNN to detect and classify faults for condition monitoring of a motor setup. This method achieves high accuracy rates and low computational complexity due to the structure of the CNN model.

Janssens et al. [69] propose a feature learning approach based on convolutional neural network (CNN) for fault detection of several faults in rotating machinery. Feature learning refers to the process of transforming raw data into a data format in an appropriate form for the intended task. This transformation is done automatically by the neural network and not by experts, as in feature engineering. The proposed approach consists of two pipelines. The first pipeline determines if the data shows rotor imbalance. This is achieved by feature extraction and logistic regression. The second pipeline shall detect four different fault types with a CNN. For comparison, the second pipeline is also implemented with feature extraction and random forest method and SVM method with different kernels. The evaluation results show that random forest performs best for the approaches without feature learning, but the approach with feature learning based on CNN performs better than the random forest approach.

#### 3.2.3. Sparse Representation Classification

Sparse representation classification (SRC) is proposed by Wu et al. [72] for fault classification. As the transfer of a multi-class classification problem into several binary classification problems is a time-consuming process, the SRC method uses training data sets with class labels to build a dictionary. To classify a new data sample, sparse representation is performed. The model finds those entries of the dictionary which represent the new data sample best. For dictionary entries, which represent sparsely, the new data sample with the smallest error defines the new data sample’s class [84]. The approach is validated by an experiment on the Tennessee Eastman process.

#### 3.2.4. Support Vector Machines

Support vector machines (SVMs) are used by Laouti et al. [73] to detect 10 different fault types in wind turbines. These include sensor faults, actuator faults, and system faults. For each fault type, one model is trained. All models use the Gaussian radial basis function as kernel function, but each fault obtains different vectors used for classification. Their method is validated by application on the data of a real wind sequence, and the results show that 6 out of 10 faults could be detected with acceptable accuracy. Imbalanced data influences the performance of the SVM method significantly, but this can be handled by assigning weights to training samples or by oversampling of training samples of minority classes [47].

Yan et al. [74] propose a hybrid approach for fault classification using an autoregressive model with exogenous variables (ARX) for data pre-processing and SVM with Gaussian radial basis function as kernel function for the classification of five different fault types and normal state. The ARX model is used to remove variable correlations, hence reducing the used variables. The model is suitable for online applications as parameters are estimated recursively. However, the method is not suitable for applications where faults need to be characterized in a short amount of time, because a time interval is required to detect the impact of a fault on the parameters. The study used a time interval of two minutes. The SVM model adopts the one-against-all algorithm, where an originally two-class SVM classifier is constructed for all pairs of fault classes, respectively. The results are validated by comparing them with several other approaches using variations of data pre-processing and SVM.

#### 3.2.5. Decision Trees

Decision trees are used additionally to SVMs by Demetgul [75] to identify 12 fault types occurring in a didactic modular production system. Several kernels and decision tree methods are used. The test setup provides signals of 8 sensors during normal and fault operation. The results show that SVM achieves test accuracy of 100% for all used kernels except for the sigmoid kernel (52.08%). The decision tree models achieve test accuracy of 100% as well, except for the decision tree trained by Chi-square automatic interaction detection (CHAID) method (95.83%).

#### 3.2.6. Tree-Structured Fault Dependence Kernel

Tree-structured fault dependence kernel (TFDK) is an approach that can be described as a hierarchical version of a large-margin SVM. This method includes fault dependence information into the learning algorithm by assigning tree-structured labels to training data, representing their fault type and severity level. Li et al. [80] use a TFDK as a learning method for the classification of real-time sensor measurements into fault types of building cooling systems and severity levels. The sample training and test data from several fault data sets reduce the data imbalance. The approach is evaluated in a cyber-physical test environment equal to the one used in Li, Hu, and Spanos [34]. Results are compared to other methods like multi-class SVM, decision tree, and neural network, which are all outperformed by the proposed tree-structured method regarding classification accuracy.

#### 3.2.7. Linear Discriminant Analysis

Linear Discriminant Analysis (LDA) is a supervised learning technique that reduces the dimensionality in data while obtaining a maximum amount of information by combining state indicators into so-called discrimination functions [85]. Li, Hu, and Spanos [34] propose a two-stage method for fault classification and diagnosis of building chillers based on LDA. Their method formulates a multi-class classification problem, including seven faulty states and the normal condition. Eight data sets for each fault type and normal condition are well separated in a lower-dimensional space produced by LDA, and each of the data sets forms a cluster. New monitored data will be put into the cluster with the lowest Manhattan distance between the data point and cluster center. An unknown fault is identified if the distance is higher than a threshold. As most sensors produce data continuously, the training data set can be updated in case an unknown fault is detected. The same algorithm is used to classify monitored data into fault severity clusters, where each cluster represents a defined severity level. The approach is evaluated with an experiment on an integrated cyber-physical test environment.

### 3.3. Fault Prioritization

Fault prioritization is the process of deciding which fault must be eliminated first to reduce the overall fault impact on the production output, especially loss of production. To the best of our knowledge, there are no automated methods proposed to prioritize faults in industrial production processes in literature. Papers that handle fault or maintenance prioritization request that fault types are prioritized by risk or severity levels beforehand by experts [34,80,86]. The assignment of newly discovered faults to these prioritized fault types results in an indirect prioritization. The method Failure Mode and Effects Analysis (FMEA), presented in Section 3.3.1 shows how experts can be supported in their decision process of defining the risk and severity levels for the different identified fault types. In the end, the defined priority levels can be assigned to the fault types, and fault classification as shown in Section 3.2 can be used to prioritize faults indirectly.

As an outlook, fault prioritization is also part of other research fields, e.g., software development. Here, bugs or faults will be categorized and prioritized during development. With this aggregated knowledge beforehand, the training of the classifiers is possible in order to rank novel faults, e.g., with natural language processing [87], SVM [87,88,89], Naïve Bayes [88], k-Nearest Neighbors [88,89] and neural networks [88,89,90]. A similar approach is conceivable if the huge amount of data is partially presented and annotated by domain experts in a production site. As a result, the ideas of fault prioritization in software development can also be used to prioritize faults in the Industry 4.0 domain in the future.

On the contrary, for software development ideas, FMEA is already used for fault prioritization in the production domain. For this reason, we narrow the FMEA methods subsequently.

#### 3.3.1. Failure Mode and Effects Analysis

FMEA is an effective method for failure analysis, identification, and classification, as well as risk assessment of these faults [91,92]. This method originated in the 1960s and was used in the aerospace industry for solving problems of quality and reliability of products [93]. Subsequently, the method was also used in the production industry as a risk assessment tool to increase the quality and stability of systems [93,94,95,96]. FMEA has also been used for CPSs [92,97,98]. The representation of FMEA in literature has risen, especially since 2013 [94,95,96]. Some papers are using Failure Mode, Effect and Criticality Analysis (FMECA) if a criticality analysis is included [99,100].

With the FMEA method, several product development steps, including product manufacturing, can be analyzed separately, and potential fault types can be identified and assessed regarding their risk and impact on further manufacturing steps. In product manufacturing, fault types depend on the architecture, the characteristics, and functionalities of the production line so that experts are needed to identify dependencies of the used machines and their potential fault types [94]. After the definition of Risk Priority Numbers (RPNs) for all fault types, an assignment of RPNs to the faults is performed. The RPN enables the comparison of the risks of various machine faults. In the original FMEA, the RPN depends on the numerical assessment, with values 1 to 10, of the fault’s severity, occurrence, and detection, where detection gives the probability that a fault is not detected until a failure occurs that impacts the customer [95,101].

However, the RPN experienced some criticism in literature due to some disadvantages that cause the resulting prioritization to be inaccurate [95,102]. The critical shortcomings can be briefly summarized as: (1) the usage of three simple factors (severity, occurrence, detection) does not guarantee that faults with equal risks get an equal RPN and faults with different risks get different RPNs [100,103,104], (2) the difference between RPNs of two faults does not represent their actual risk difference [103], (3) the three risk factors are considered to be equally important, so factor weights are not considered [100,103], and (4) the subjectivity of the RPN factors, caused by the subjectivity of the experts who define the factors for all fault types, is not represented in the resulting RPN and leads to uncertainty [92,100,103].

This criticism caused researchers to develop extensions for the calculation of RPNs so that there are now various algorithms used to define the RPN of faults. An overview of these extensions and algorithms is displayed in Table 3. One simple adaptation of the RPN is the consideration of additional risk factors, which leads to a diversification of resulting risk numbers. Examples for additional risk factors are “expected cost of failure” [91], “environmental factors” [92], and further “economic impact” [92,105]. Additional risk factors work against points of criticism (1) and (2). Another extension comparable to additional risk factors is the splitting of one risk factor into several sub-risk factors. This leads to a more precise definition of the original risk factors because more diverse aspects are considered. An example is the partition of the severity risk factor. To define the severity of a fault, both technical and economic aspects should be considered [105,106].

To handle the subjectivity of defining the risk factors, some authors suggest using fuzzy logic as an extension of FMEA [112,117,119]. In these approaches, the risk factors are defined via linguistic variables like low, medium, and high instead of numerical variables [111,112,113]. Those variables are then ranked with fuzzy numbers, which means that the number has one assigned value and multiple values with probabilities, so that the severity can be ranked as, e.g., {30%: 3, 50%: 4, 20%: 5} instead of using one numerical value on the scale of 1 to 10 [111,112].

Multi-criteria decision-making (MCDM) methods are also widely used to overcome the shortcomings of original RPNs. MCDM is a branch of operations research that supports experts in their decision-making process [120]. Examples for MCDM methods are the analytic hierarchy process (AHP) [108,117,118] and the technique for the order of prioritization by similarity to ideal solution (TOPSIS) [113,117,119].

## 4. Discussion

The developed process of fault handling, as illustrated in Figure 1, begins with data collection, pre-processing, and feature handling. Research provides numerous attempts to handle these steps. For each of the described fault handling methods, other types of preparations are necessary, denoting why it has not been described in this review in the first instance. However, fault handling requires representative data to operate effectively and generate sensible output. So while following the described fault handling process, sufficient work should be invested into accurate data. Only very few data sets in the context of industrial applications and smart manufacturing are available for open access [17], which exacerbates validation of the defined fault handling process significantly. Therefore, validation shall be fetched in a later stage.

With the increase of computational resources at the beginning of the 21st century, the focus seems to shift from model-based to data-driven fault detection methods. Due to Industry 4.0 and further digitalization of processes, the complexity of manufacturing systems also enhances, making model-based and knowledge-based approaches challenging to handle. A more significant part of fault detection has been carried out by variants of neural networks and deep learning regarding data-driven methods. We see a trend that neural networks and deep learning will be used primarily in the future, as computational resources are not that critical anymore. However, we showed that other promising methods exist, which can be used for some use cases in the industry. Ensemble methods tend to improve the results in various ways compared to single methods. We have given a combination of kNN and Naïve Bayes, which reduces non-informative data sensitivity. Especially TEDA and OS-LSSVM can detect faults very fast, which is crucial for real-time applications. We assume that a combination of deep learning and other methods to an ensemble would speed up the fault detection process by providing the benefits of deep learning.

The situation for fault classification behaves very similarly compared to fault detection. Increased computational resources facilitate and promote neural networks and deep learning. Autoencoders became a standard unsupervised learning method for fault classification. Additionally, fault classification requires fault detection beforehand, in which variants of neural networks will be implemented more often. Some papers also showed that one deep learning algorithm can handle a combination of fault detection and classification [121,122]. So further applications on fault classification by neural networks and deep learning are likely to be implemented. Beneath deep learning, SVMs are still broadly used for fault classification, often but non-exclusive for supervised learning tasks. Some tree-like algorithms such as decision trees and random forests have been applied for fault classification, which often explains its results more naturally than deep learning. TFDK provides an interesting approach to classify faults by using severity levels. This algorithm may also be used in fault prioritization, which significantly fastens the fault handling process. To the best of our knowledge, this method has not been implemented in another context, so it may still be an open issue for future research on fault handling.

Fault prioritization in an industrial context is difficult to achieve, as non-statistical algorithms are rare. FMEA is an effective method extended frequently to reduce the impact of criticized disadvantages. The method has proven its operational capability in various industrial environments. However, this method is statistical and expensive regarding both time and work. The method cannot be automated, as it requires a high degree of knowledge within the domain. The first attempts to design automated fault prioritization have been made, but not in Industry 4.0. We see a vast potential to transfer knowledge from other disciplines into the manufacturing domain. This is crucial as factories tend to become more complex in the process of digitalization, and only efficient prioritization prevents production outages.

As discussed earlier, some methods cover more than one phase of the mid-tier process. Nevertheless, no known method covers all three steps of fault detection, fault classification, and fault prioritization. This is basically due to missing algorithms for fault prioritization. However, the chances are that a methodology can be developed with automated prioritizing methods, which covers the whole mid-tier fault handling process in the future. Such a methodology would be a step forward, especially with fully automated smart manufacturing systems.

For fault amendment, innumerable methods exist, as these methods strongly depend on the results of the preceding mid-tier process and the experience of the operator’s personnel. So, generalization of this process step is not easy to achieve and, therefore, will not be covered in this paper. An automated approach regarding fault amendment is also challenging to realize. With new knowledge regarding additional non-statistical fault prioritization methods with certain automated aspects, fault amendment needs to be re-evaluated and possibly leaves additional prospects for future research.

## 5. Conclusions

We structured fault handling in the context of Industry 4.0 by defining the process to scope future discussions in the research field. In addition, the requirements for fault handling are defined. Moreover, a survey is provided, which was structured by the presented process. Here, the survey is focused on the mid-tier fault handling processes. Only selected examples for each category are provided for each sub-process because various types and architectures exist for each method. Therefore, the scope of this work is to provide guidance and an overview of current state-of-the-art fault handling techniques rather than to claim completeness. The categories of each sub-process are designed to identify research for further reading. Evaluated research was not always in the context of CPSs and may miss certain information about the used evaluation environment, which made it impossible to choose publications according to the presented requirements. Future attempts should point out how methods can be assessed to meet the requirements of industrial processes more precisely. The research also showed that requirements differ for each industrial plant due to high specialization and possibilities in configuration. Therefore, methods should be validated in the area of the industrial process before implementation.

We have also identified techniques outside of Industry 4.0 that can give impetus to the fault handling process, especially fault prioritization, in the future. Methods from the field of software development may be useful to classify and prioritize novel faults. Compared to fault detection and classification methods, automated methods of fault prioritization could not be identified in the literature. Here, many authors state that experts are needed to prioritize faults due to a high individuality of the faults. Therefore, the prioritization of faults solely accomplished by statistical learning methods is unlikely. FMEA was presented in this paper as a method to support experts in their decision-making regarding machine risks. Support methods for experts should be further investigated and optimized, for example, by more thorough experts interviews. Another unexplored path is fault amendment, and the first promising work has been done with the automated reconfiguration of plants in a failure state, but the field beholds much potential. Future work of our defined process encompasses the definition and validation of non-statistical fault prioritization methods in Industry 4.0. With an accurate data set of industrial origin, the described process can be validated and adapted if necessary. Finally, the development of a methodology that covers the whole mid-tier fault handling process for an industrial plant can be performed. The process and the overview given in this work should be seen as a starting point for the definition of fault handling in the Industry 4.0 domain. Each part is open to future research and may provide guidance.

## Figures and Tables

**Table 1:** Overview of used methods for fault detection with references.

Method	Details	References
Neural Network	Self-organizing map	[36]
	ANN	[37,38]
Random Forest	Classification Problem (normal, fault)	[39]
k-Nearest Neighbors (kNN)	Ensemble method based on kNN with random forest k-means for feature selection	[40]
Naïve Bayes classifier	Ensemble method based on Naïve Bayes classifier with random forest k-means for feature selection	[40]
Kernel PCA	Training on only normal data points and using threshold for fault detection	[19,41,42]
TEDA (Typicality and Eccentricity Data Analytics)	Unsupervised algorithm, no previous knowledge needed; detects outliers as faulty data samples	[3,43,44,45]
Improved Support Vector Machines (SVM)	OS-LSSVM uses a sparsity component to increase the prediction speed of sensor values; fault is detected in case of high residual error	[46,47,48]

**Table 2:** Overview of used methods for fault classification with references.

Method	Details	References
k-means	Clustering method to identify fault types	[1,2,65]
Gaussian Mixture Model	Clustering method to identify different distributions	[1,2]
Fuzzy-c-means	Extension of K-means with fuzzy variables	[2]
	Autoencoder	[49,66]
Neural Network	ANN with softmax layer	[37,67,68]
	CNN-based feature learning	[69,70,71]
Sparse Representation Classification (SRC)	Performs classification based on sparse representation of training data	[72]
SVM	Two-class classifier or pair-wise classifiers for multi-class classification	[51,67,69,70,73,74,75,76,77,78]
Decision Tree	Classification with QUEST, C&RT, C5.0, and CHAID methods	[75]
Random Forest	Random Forest with feature extraction	[69,79]
Tree-structured fault dependence kernel	Hierarchical large margin SVM	[80]
Linear Discriminant Analysis	Uses distance metrics to assign classes	[34]

**Table 3:** Overview of extensions for RPN calculation.

Extension	Details	References
Additional risk factors	e.g., expected cost, cost of failures, weight of corrective actions, uncertain risk factors, environmental factors, economic safety	[91,92,105,107,108,109,110]
Usage of sub-risk factors	e.g., severity levels from various perspectives like technical or economical	[104,105,106]
Fuzzy variables	Fuzziness used in variables to represent uncertainty and imprecise risk factors	[111,112,113]
Multi-criteria decision methods	Defining risk based on multiple conflicting criteria	[108,113,114,115,116,117,118,119]
